# Impact of Deoxycholic Acid on Oesophageal Adenocarcinoma Invasion: Effect on Matrix Metalloproteinases

**DOI:** 10.3390/ijms21218042

**Published:** 2020-10-28

**Authors:** Fran Quilty, Anne-Marie Byrne, John Aird, Sheeren El Mashad, Adolfo Parra-Blanco, Aideen Long, John F Gilmer, Carlos Medina

**Affiliations:** 1 School of Pharmacy and Pharmaceutical Sciences, Trinity Biomedical Sciences Institute, Trinity College Dublin, 2 Dublin, Ireland; quiltyf@gmail.com (F.Q.); amb@tcd.ie (A.-M.B.); gilmerjg@tcd.ie (J.F.G.); 2 School of Medicine, Trinity Translational Medicine Institute, Trinity College Dublin, Trinity Centre for Health Sciences, St James’s Hospital, 8 Dublin, Ireland; longai@tcd.ie; 3 Department of Histopathology and Morbid Anatomy, School of Medicine, Trinity College Dublin, 2 Dublin, Ireland; ja@tcd.ie; 4 Cork Cancer Research Centre, BioSciences Institute, University College, T12 YN60 Cork, Ireland; selmashad@yahoo.ie; 5 National Liver Institute, Menoufiya University, Shebin El Kom, Egypt; 6 Department of Gastroenterology, NIHR Nottingham Biomedical Research Centre, Nottingham University Hospitals NHS Trust and University of Nottingham, Nottingham NG11 8NF, UK; parra.blanco@gmail.com

**Keywords:** deoxycholic acid, matrix metalloproteinases, mmp-10, oesophageal adenocarcinoma

## Abstract

Bile acids (BAs) have been implicated in the development of oesophagitis, Barrett’s oesophagus and oesophageal adenocarcinoma (OAC). However, whether BAs promote cancer invasiveness has not been elucidated. We evaluated the role of BAs, in particular deoxycholic acid (DCA), in OAC invasion. Migration and invasiveness in untreated and BA-treated oesophageal SKGT-4 cancer cells were evaluated. Activity and expression of different matrix metalloproteinases (MMPs) were determined by zymography, ELISA, PCR and Western blot. Finally, human OAC tissues were stained for MMP-10 by immunohistochemistry. It was found that SKGT-4 cells incubated with low concentrations of DCA had a significant increase in invasion. In addition, MMP-10 mRNA and protein expression were also increased in the presence of DCA. MMP-10 was found to be highly expressed both in-vitro and in-vivo in neoplastic OAC cells relative to non-neoplastic squamous epithelial cells. Our results show that DCA promotes OAC invasion and MMP-10 overexpression. This study will advance our understanding of the pathophysiological mechanisms involved in human OAC and shows promise for the development of new therapeutic strategies.

## 1. Introduction

Common oesophageal complications of gastro-oesophageal reflux disease (GORD) include reflux oesophagitis and Barrett’s oesophagus (BO), which is a known risk factor for the development of oesophageal adenocarcinoma (OAC). It is thought about 10% of all GORD patients develop BO [1,2]. Population-based studies have found the risk of OAC to be 30 to 60 times greater in BO although only 0.2–0.5% of patients with BO will develop OAC [2,3]. Proton pump inhibitors (PPIs) are very effective for healing reflux oesophagitis as they are the most potent inhibitors of gastric acid secretion. However, the widespread use of PPIs has not affected the incidence of OAC. Therefore, low pH does not seem to be the principal pathogenic factor in OAC development and content of refluxate other than acid might contribute to the progression of cancer within BO.

Bile acids (BAs) are steroid-like amphiphilic substances which assist in the absorption of dietary fats and lipids [4]. In humans, the predominant BAs are cholic acid (CA), chenodeoxycholic acid (CDCA) and deoxycholic acid (DCA). The primary BAs, CA and CDCA, are synthesized in the liver and stored in the gallbladder. Formation of the secondary BAs, DCA from CA and lithocholic acid (LCA) from CDCA, is catalysed by available 7α-dehydroxylase expressing bacteria [5]. Despite the fact that BAs play an important role in biological functions, they have been implicated in pathological conditions such as oesophagitis, BO and OAC development [6,7]. Conjugated and free BAs are found in the refluxate of GORD patients and their concentration correlates with the degree of tissue damage. However, the precise contribution of refluxed BAs to the pathogenesis of GORD and its complications remains unclear. It has been previously shown that BAs can stimulate oesophageal squamous epithelial cells and Barrett’s epithelial cells to produce proinflammatory cytokines such as IL-8 through the NF-κB pathway [8,9,10]. Bile acids could cause an increase in reactive oxidative species (ROS) that leads to DNA damage [11]. Bile acids also cause oesophageal squamous cells to express Cdx2 [12] which has been implicated in the early development of metaplasia in oesophageal cells. Notch signalling is directly related to Cdx2 and the induction of Cdx2 coincides with atonal homolog 1 (ATOH1) activation and the resultant differentiation of stem cells to goblet cells [13]. Bile acids also induce the expression of MUC2 which is a mucin gene highly expressed by goblet cells in BO [14,15]. This is supported by animal studies in which direct oesophageal BA exposure leads to severe oesophagitis, BO and OAC [16,17].

The matrix metalloproteinases (MMPs) are a family of enzymes that digest components of the extracellular matrix (ECM) and are involved in cancer spreading and invasion [18,19,20]. Increases in MMP-1, MMP-2, MMP-7, MMP-9 and MMP-10 expression have all been linked to progression of normal oesophageal tissue to OAC [21,22,23,24,25,26] but the mechanism underlying what causes the increase in expression is currently unknown. It has been previously shown that BAs can promote MMPs that in turn enhance colon cancer invasion and metastasis [27]. In this study, we hypothesised that in addition to being a causative factor for the occurrence of OAC, BAs, in particular DCA, may increase oesophageal tumour cell invasion and metastasis through an up-regulation of MMPs. Therefore, our first aim was to study the effect of DCA on the invasive capacity of SKTG-4 cells, a human OAC cell line. Next, we studied the effect of BAs on the expression and activity of MMPs in SKTG-4 cells. Finally, we performed immunohistochemistry on OAC patient tissue to study the expression and distribution of a selected MMP.

## 2. Results

### 2.1. Bile Acids Promote OAC Cell Invasion

**Figure 1:**
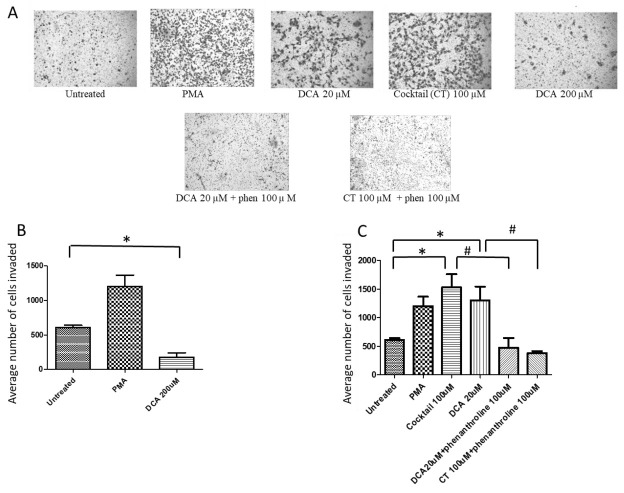
Effect of bile acids (Bas) on cell invasiveness. (**A**) Representative images of invasion assays in untreated SKG-4 cells and treated cells (PMA 100 µM, deoxycholic acid (DCA) 20 µM, cocktail (CT) 100 µM, DCA 200 µM, DCA 20 µM + phenantroline (phen) 100 µM and CT 100 µM + phen 100 µM). (**B**) and (**C**) The corresponding bar graph showing the results of invading cells (* *p* < 0.05 versus untreated cells; # *p* < 0.05 versus cocktail (CT) 100 μM and DCA 20 μM). *n* = 3 for each group.

To examine the effect of BAs, in particular DCA on cancer cell invasion, the ability of SKGT-4 cells to invade was assessed using a matrigel invasion assay in the presence of DCA at high concentration (200 μM), at low concentration (20 μM) or with a 100 μM cocktail of BAs reflective of the composition of patient refluxate. It was found that the number of invading SKGT-4 cells was significantly increased when cells were treated with DCA (20 μM) and the cocktail (100 μM) as seen in Figure 1A. In contrast, DCA at high concentration (200 μM) reduced the number of invading SKGT-4 cells (Figure 1A,B). In addition, pre-treatment with the broad spectrum MMP inhibitor phenanthroline (100 μM) prior to DCA/cocktail also reduced SKGT-4 cells invasiveness (Figure 1C).

### 2.2. DCA at High Concentration Reduces Cell Viability

As DCA 200 μM was able to reduce the number of invading SKTG-4 cells, MTT assays were carried out to study whether BAs exerted any effect on cell survival to rule out the possibility that inhibition of cancer invasion was due to cell death. As shown in Figure S1, DCA (20 μM) and cocktail (100 μM) did not reduce cell survival compared to untreated cells. However, DCA at a concentration of 200 μM significantly reduced cell survival. Therefore, DCA at 20 μM was used for the next experiments and because it is more pathophysiologically relevant since in many cases DCA is present at a very low concentration in refluxate. 

### 2.3. DCA Does Not Exert Any Effect on 2D Cell Migration

**Figure 2:**
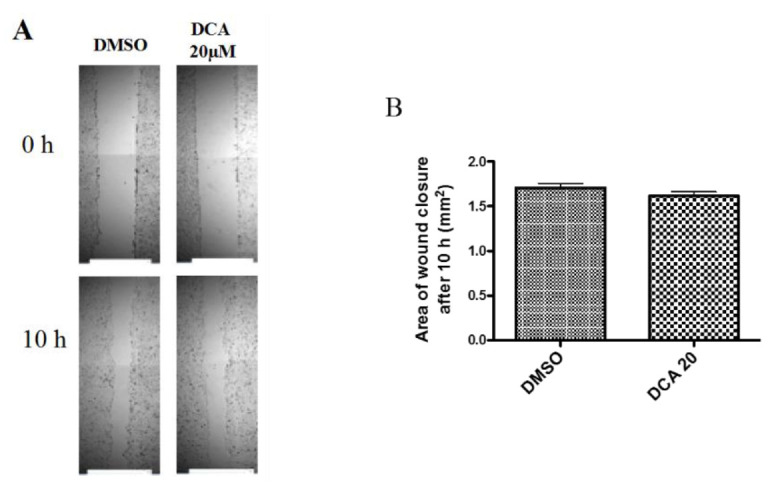
Effect of DCA on 2-D migration. (**A**) Images taken on the Cellavista of the scratch wound at time 0 and after 10 h in DMSO (0.1%) and DCA (20 μM)-treated cells. (**B**) The corresponding bar graph showing the results of the migration assay. *n* = 3 for each group.

In order to determine whether the increase in invasion was associated with an increase in cell migration, SKGT-4 cells were treated with DCA (20 μM) and a simple scratch wound assay was performed. The area of the scratch at 0 h was imaged to ensure that both scratches were comparable at the beginning of the assay as seen in Figure 2. The areas were then compared at 10 h and no significant difference was found between the DMSO treated well and the DCA (20 μM) treated well.

### 2.4. Profiling MMP Expression and Activity in Oesophageal Epithelial Cell Lines Representing the Normal-BO-OAC Sequence

**Figure 3:**
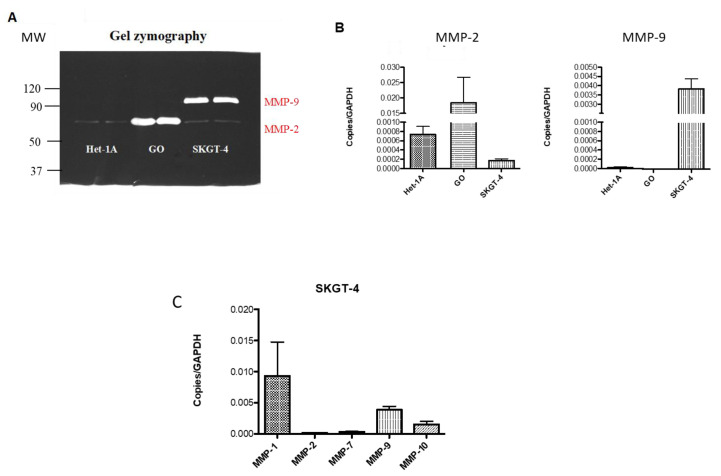
Basal activity and expression of matrix metalloproteinases (MMPs) in oesophageal cells. (**A**) Representative zymography from non-stimulated Het-1A, GO and SKGT-4 cells and quantitative analysis (**B**,**C**). Expression profile of different MMPs at gene level in non-stimulated SKGT-4 cells. *n* = 3 for each group.

After ruling out 2-D migration, we next studied the expression of MMPs in an attempt to determine the mechanism of increased invasion mediated by DCA. Before measuring the effects of DCA on MMP activity, oesophageal epithelial cell lines from across the normal-BO-OAC sequence were tested for basal MMP activity. Het-1A cells represent squamous epithelial cells, GO cells represent dysplastic BO and SKGT-4 cells represent OAC. Using gel zymography levels of active MMP-2 and MMP-9 secreted from untreated Het-1A, GO and SKGT-4 cells were examined. The OAC SKGT-4 cells were found to secrete the highest concentration of active MMP-9 and the dysplastic GO cell line secreted the highest concentration of MMP-2 (Figure 3A). MMP-2 and MMP-9 mRNA levels were then measured in untreated Het-1A, GO and SKGT-4 cells to determine whether a direct correlation could be found with MMP activity. The dysplastic GO cell line was found to have the highest basal level of MMP-2 mRNA expression and the SKGT4 cells had the highest basal level of MMP-9 mRNA expression (Figure 3B). These results demonstrate the relative basal expression levels are similar to the secreted active MMP levels detected using gel zymography. After establishing the differing MMP-2 and MMP-9 activity and expression over the stages of OAC development a more detailed examination of a panel of MMP expression levels was performed on the most relevant cell line for invasion and migration, the OAC SKGT-4 cell line. Basal MMP-1 mRNA expression in SKGT-4 was found to be the more highly expressed than MMP-2, MMP-7, MMP-9 and MMP-10 mRNA as shown in Figure 3C. The MMP-1 mRNA expression was 2.4-fold greater than MMP-9 and 6.0-fold greater than MMP-10 in SKGT-4 cells, which are the next most highly expressed MMPs from this panel. MMP-2 and MMP-7 mRNA basal levels were found to be much lower.

**Figure 4:**
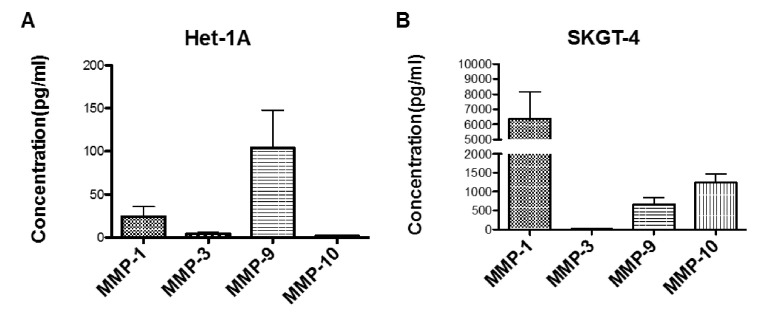
Basal protein expression of MMPs in oesophageal cells. Quantification of secreted levels of MMP-1, MMP-3, MMP-9 and MMP-10 in non-stimulated Het-1A cells (**A**) and non-stimulated SKGT-4 cells (**B**). *n* = 3 for each group.

To determine the importance of MMP-1, MMP-9 and MMP-10 in oesophageal cancer, a comparison of MMP expression was made between untreated Het-1A cells and untreated SKGT-4 cells. This insight could indicate changes in MMP levels involved in the progression of oesophageal cancer. It was found that levels of MMP-1, MMP-9 and MMP-10 were increased 265-fold, 6-fold and 800-fold, respectively in the SKGT4 OAC cells compared to the Het-1A squamous epithelial cells as shown in Figure 4A,B. The high concentration of secreted MMP-10 from SKGT-4 cells correlates with the corresponding mRNA expression levels of the cell line. 

### 2.5. Measurement of Effects of BAs on MMP mRNA Expression in SKGT-4 Cells

**Figure 5:**
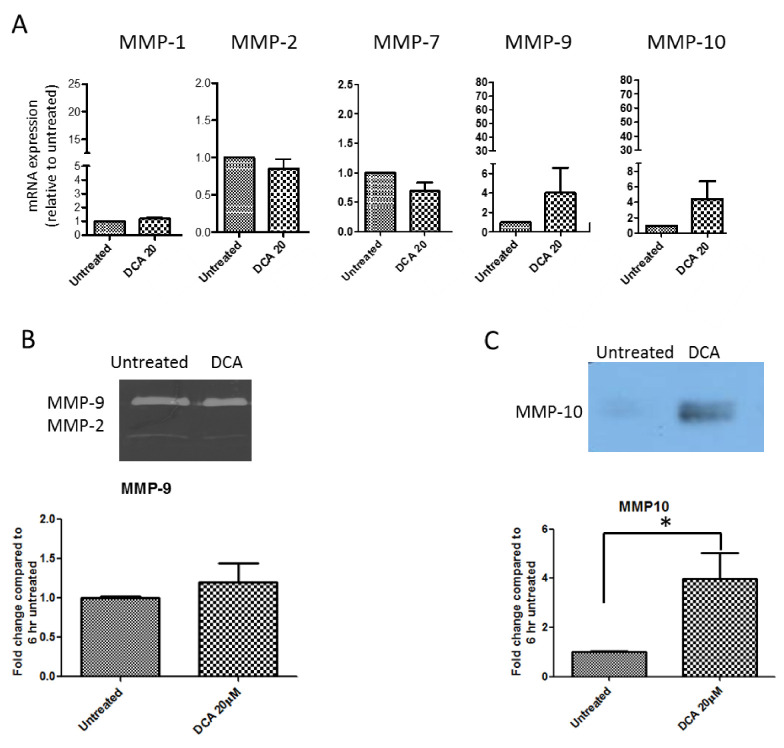
Activity and expression of MMPs in DCA-stimulated SKGT-4 cells. (**A**) Quantification of MMP-1, MMP-2, MMP-7, MMP-9 and MMP-10 at gene level in untreated and DCA (20 μM)-treated cells. (**B**) Representative zymography and quantification of MMP-9 activity in untreated and DCA (20 μM)-treated cells. (**C**) Representative Western blotting and quantification of MMP-10 in untreated and DCA (20 μM)-treated cells (* *p* < 0.05 versus untreated cells). *n* = 3 for each group.

As the MMP inhibitor prevented the BA-induced increase in SKGT4 invasion, we tested the impact of DCA (20 μM) on the mRNA expression of several MMPs. As shown in Figure 5A, DCA was able to induce a 4-fold increase in MMP-10 and 3-fold increase in MMP-9 mRNA. However, MMP-1, MMP-2 and MMP-7 mRNA levels were not affected by DCA. 

### 2.6. DCA Up-Regulates MMP-10 Protein Expression in SKGT-4 Cells

As DCA produced an increase in MMP-9 and MMP-10 mRNA expression we next studied MMP-9 protein activity and MMP-10 protein expression upon incubation with DCA at 20 μM. As shown in Figure 5B, there was not a significant increase in MMP-9 activity in DCA stimulated-SKGT-4 cells. However, DCA was able to up-regulate MMP-10 protein as shown in Figure 5C.

### 2.7. MMP-10 Is Over-Expressed in OAC Patient Tissue

**Figure 6:**
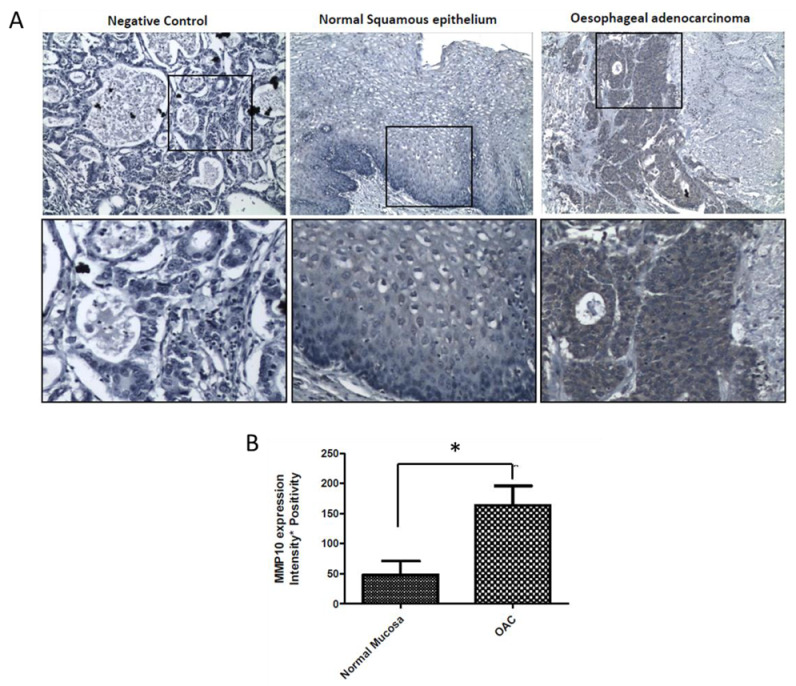
(**A**) Representative images of MMP10 immuno-histochemical staining of normal squamous epithelium (*n* = 4 patients) and oesophageal adenocarcinoma (OAC) tissue (*n* = 8 patients). A negative control was included where the primary antibody was replaced with normal goat serum during the immunohistochemistry staining. (**B**) Quantitative analysis (* *p* < 0.05 versus normal squamous epithelium).

MMP-10 expression was investigated using tissue from patients with OAC. Normal mucosa was obtained adjacent to the tumour. Areas of carcinoma were identified by a trained pathologist. Low levels of MMP-10 expression were observed in non-neoplastic oesophageal squamous epithelium whereas increased expression was observed in OAC tissue (Figure 6).

## 3. Discussion

There is increasing evidence that BAs, in particular DCA play an important role in oesophagitis, BO and OAC development. In this study we provide pioneering evidence that DCA has the capacity to promote OAC invasion through the induction of MMPs. First, we studied whether BA cocktail and DCA were able to induce 3D migration in SKGT-4 cells. From studies in the literature, it is clear that the constituents of refluxate vary drastically in patients with GORD [28]. The debate exists over what concentration of free DCA is present in the refluxate. Two of the main studies that quantified the levels of BAs in the refluxate of patients with GORD found DCA levels of 282 μM [28] and 115 μM [29], which were both high enough to induce a pro-inflammatory response. Therefore, BA cocktail at 100 μM and DCA at 20 and 200 μM were tested. 

We found that the cocktail and DCA at low concentration promoted OAC invasion. For this purpose, we used a matrigel assay which has been verified by showing that the more metastatic cell lines penetrate through the basement membrane to a greater degree and non-metaplastic cells do not invade through the membrane whatsoever [30]. When a higher concentration of DCA was used, the opposite results were found. Therefore, we next studied whether BAs could cause cell death and found a reduced cell viability observed for this concentration using the MTT assay. Our results are in agreement with a previous work carried out on colon cancer cells [31]. In that study, the invasion of SW480 cells through a matrigel was significantly increased when treated with DCA (5 μM) along with the ability of the cells to form colonies. However, treatment with higher concentrations (100 μM) reduced the colon cell proliferation and inhibited colony formation in the matrigel. Those effects induced by low concentration of DCA were inhibited by the blocking of uPAR with an antibody [31].

Next, we studied whether the effects of DCA on OAC invasion were due to a direct effect on cell migration. We found that DCA treatment did not play a role in 2D migration. 2D migration involves an integrin-dependent protrusion-adhesion-contraction process mediated by the actin cytoskeleton [32]. However, as DCA promoted 3D migration, that prompted us to study the expression of MMPs. Furthermore, phenantroline, a broad spectrum MMP inhibitor, was able to inhibit the number of invading cocktail- and DCA-stimulated SKGT-4 cells. 3D migration depends on tractile and adhesion forces but also proteolytic activity of MMPs [33]. Matrix metalloproteinases are enzymes that play a key role in cancer development and they have been extensively implicated as crucial factors in tumour cell migration and invasion [34,35]. In this study, we profiled different MMPs in oesophageal epithelial cells. Basal MMP-1 mRNA expression in SKGT-4 was found to be the most highly expressed, followed by MMP-9 and MMP-10. This correlates with reports of the importance of MMP-1 in OAC in the literature as a strong association between high MMP-1 expression and positive lymph node metastases has been shown [22]. We also found that MMP-1, MMP-9 and MMP-10 are over-expressed in SKGT-4 cells compared to Het-1A cells indicating changes in MMP levels as a model of the progression of OAC. Our results are in agreement with previous human studies where protein expression of MMP-1 and mRNA expression of MMP-3, MMP-7, MMP-10 and TIMP-1 found in tissue samples were significantly increased in the progression from BO to OAC [26].

There has been some direct evidence in the literature of BAs increasing MMP expression/activation. A study on HT-29 colon cancer cells showed that exposure to DCA in concentrations ranging from 20 μM to 80 μM resulted in an increased expression and activation of MMP-9 in a concentration-dependent manner [36]. In addition, it has been shown that LCA promoted invasion in Caco-2 cells by increasing the secretion of MMP-2 [27]. Therefore, we studied the effects of DCA on MMPs at gene and protein level. In this current study, DCA was not able to induce a significant increase in both gelatinases, MMP-9 and MMP-2. However, it was found that DCA was able to induce an up-regulation in MMP-10 as detected by q-PCR and Western blot. Our results are in agreement with a previous study where SKGT-4 cells exposed to DCA (300 μM) had an increased MMP-10 mRNA expression, whereas in Het-1A cells the mRNA expression of MMP-10 was downregulated by DCA [37]. MMP-10 has been shown to degrade various components of the ECM and is considered as collagenase-related connective tissue-degrading MMP. However, the importance of MMP-10 on metastasis in oesophageal cancer is less reported in the literature. In fact, over-expression of MMP-10 has been previously been reported in patients suffering from oesophageal squamous cell carcinoma and associated with tumour size [38] and poor survival [39]. In order to investigate further the involvement of MMP-10 in OAC, we therefore conducted MMP-10 staining in OAC patient tissue. We found that MMP-10 is over-expressed in OAC tissue compared to normal squamous epithelium which is reflective of our in-vitro observations and indicates the translational relevance of this study. MMP-10 may promote tumour cell invasion in-vivo. In summary, we have demonstrated that DCA promotes invasion in OAC SKTG-4 cells. Indeed, DCA treatment was able to up-regulate MMP-10 at protein and gene level. These in-vitro observations were confirmed in human OAC tissue.

These findings provide new insights into the potential role for DCA in promoting invasion in oesophageal cancer through its effects on MMP-10 expression. This re-enforces the need to develop a therapeutic targeting BAs to prevent oesophageal cancer development, progression and invasion.

## 4. Materials and Methods

### 4.1. Cell Culture

A human OAC cell line, SKGT-4, was obtained from the European Cell Culture Collection (Salisbury, UK). SKGT-4 cells were cultured in RPMI 1640 medium supplemented with 10% heat-inactivated fetal bovine serum (FBS). An adherent human oesophageal epithelial cell line, Het-1A cells and, a BO cell line, GOhTRT (GO), were purchased from American Type Culture Collection (ATCC, Rockville, MD, USA). The cells were cultured in bronchial epithelial cell basal medium supplemented with triiodothyronine, insulin, transferrin, retinoic acid, hydrocortisone, human recombinant epidermal growth factor, epinephrine and bovine pituitary extract. All cells were cultured as monolayers in 75 mL culture flasks at 37 °C in a humidified atmosphere with 5% CO_2_. The cells were supplied with fresh medium and subcultured three times each week.

### 4.2. Invasion Assay

The invasion assay is useful to study the metastatic potential of tumour cells. Cells must pass through a matrigel matrix that serves as a reconstituted basement membrane and invade through an insert with 8 μm pores. The BD BioCoat Matrigel Invasion Chamber assay system (BD Biosciences, Bedford, MA, USA) was used to study the effects of BAs on cancer cell invasion. Briefly, aliquots of 500 μL of SKGT-4 cell suspension (3.2 × 10^4^ cells/500 μL serum free RPMI) in the presence or absence of BA cocktail consisted of BAs reported to be present in patient refluxate (100 μM composed of 20 μM each of TCA, GCA, GDCA, GCDCA and DCA) or DCA (20 and 200 μM) were added to the inserts and cells allowed to invade for 22 h. Phorbol 12-myristate 13-acetate (PMA, 100 ng/mL)-stimulated cells were used as a positive control. In addition, further experiments were carried out with an MMP inhibitor, phenanthroline (100 μM). After 22 h the matrigel in the chambers were removed and the non-invading cells on top of the insert were scrubbed off using a cotton bud moistened with medium. The insert was then immersed in 100% methanol for 2 min to fix the cells, followed by 10 min in eosin, then 10 min in hematoxylin to stain the cells and nuclei. They were rinsed in water before being allowed to air dry. Images of the inserts were taken using the Cellavista (Roche, Basel, Switzerland) and invading cells were counted using the ImageJ software (NIH, Bethesda, MD, USA). The data are representative of the mean number of cells invaded per field of view for the treatments indicated for *n* = 3 experiments.

### 4.3. Cell Survival

The MTT assay was used to study cell survival. The MTT assay is a colorimetric assay measuring the activity of an enzyme that converts methyl-thiazolyl-tetrazolium (MTT) bromide to a purple water-insoluble formazan [40]. The formazan is solubilised with DMSO and quantification of the amount present can be measured from its absorbance at 570 nM using a spectrophotometer. The assay has become accepted as a measure of cell viability and can be used to assess cytotoxicity of compounds. SKGT-4 cells were seeded at a density of 7.5 × 10^3^ cells per well in a nunclon 96-well plate and allowed to adhere overnight. The medium was removed in each well and either replaced with fresh media in those wells that had no pre-treatment or else serum free media with DCA (20 μM), BA cocktail (100 μM) or DCA (200 μM) for 22 h. Three hours prior to the endpoint of the experiment 20 μL of 2.5 mg/mL MTT (Sigma, St. Louis, MO, USA) was added to each well. All media was removed at the 22 h time point and the cells were immersed in 100 μL DMSO for 30 min to allow for dissolution of the formazan crystals. The absorbance was read at 570 nM using a BioTek EL ×800 absorbance micro-plate reader (BioTek, Winooski, VT, USA). All measurements were normalised to the untreated control for the time-point.

### 4.4. Assessment of the 2D Migration Using the Scratch Wound Assay

The scratch wound assay is a simple assay commonly used to measure basic cell migration parameters such as polarity, speed and persistence. Cells at the wound edge polarize and migrate into the scratch wound. SKGT-4 cells were seeded at a density of 4 × 10^4^ cells per well in a 96-well plate and allowed to form a confluent monolayer. Cell monolayers were then wounded using a p200 pipette tip. Wounds were gently washed once and medium was then replaced with complete medium supplemented with either DCA (20 µM) or with DMSO (0.1%) as a vehicle control. Wounds were imaged immediately after wounding (0 h) and at 10 h post wounding using the Cellavista Imaging platform (Roche, Basal, Switzerland). Migration was quantified by measuring the area of wound closure at 10 h post wounding.

### 4.5. MMP mRNA Expression Using Quantitative Real-Time PCR

Quantitative PCR (qPCR) was used to profile MMP expression in cancer cells and whether or not BAs had an effect on MMP gene expression. Briefly, total cellular RNA was isolated from cancer cells using RiboPure kit from Ambion (Austin, TX, USA) according to manufacturer’s protocol. The RNA was quantified on the Nano Drop 8000 (Thermo Scientific, Wilmington, DE, USA) where the quality of mRNA was evaluated by the OD260/OD280 ratio. Only samples with a ratio value of ~2.0 were used. For reverse transcription reaction, 1 µg of total RNA (High Capacity cDNA Archive Kit) was used and 10 ng of transcribed DNA was spent for each quantitative PCR reaction. As target probes, TaqMan MGB human MMP-1, MMP-2, MMP-7, MMP-9 and MMP-10 (Applied Biosystems, Dublin, Ireland) were used along with a housekeeping gene glyceraldehyde-3-phosphate dehydrogenase (GAPDH). Fold inductions were calculated using the comparative CT method as described in the ABI Prism manual using GAPDH as the internal control [41]. Gene expression levels of the MMPs were expressed as the cycle threshold (C_t_) value (MMP)/C_t_ value (GAPDH). GAPDH levels (C_t_ values) were similar across all cell lines. Values are presented as fold change in gene expression relative to the control group, which was normalized to 1.

### 4.6. MMP Activity Detection by Gelatin Zymography

To investigate MMP-2 and MMP-9 activity, gelatin zymography was carried out as previously described [42]. Briefly, gelatin zymography was performed using 8% sodium dodecyl sulphate–polyacrylamide gel electrophoresis with copolymerized gelatin (2 mg/mL). After electrophoresis, gels were washed in 2.5% Triton X-100 for 1 h (three times, 20 min each) and incubated for 24 h in enzyme assay buffer (25 mM Tris, pH = 7.5, 5 mM CaCl_2_, 0.9% NaCl, 0.05% NaN_3_). The gelatinolytic activities were detected as transparent bands against the background of Coomassie blue-stained gelatin. Bands were quantified using ImageJ software (NIH, Bethesda, MD, USA).

### 4.7. MMP Secretion Detection by Multiplex ELISA

Human MMP-Plex Ultra-Sensitive Kits (MSD, Gaithersburg, MD, USA) were used to estimate concentration of human MMP-1, MMP-3, MMP-9 and MMP-10. Cells were grown in 24-well plates until 70% confluent. The supernatant was removed from the wells and stored at −20 °C. The experiment was carried out according to the MSD protocol. The sample or standard (25 μL) was added into the well and the plate was shaken at 900 rpm for 2 h at room temperature. The plate was subsequently washed three times with PBS with tween (PBS-T) and the detection antibody solution (25 μL) was dispensed into each well. This was left shaking (900 rpm) for 2 h at room temperature. Lastly the plate was washed 3 times with PBS-T and 150 μL of 2× read buffer T was added to each well of the plate. The plate was read immediately on the Sector Imager (MSD, Gaithersburg, MD, USA) and concentrations of each MMP calculated from the calibration curves.

### 4.8. MMP Expression Detection by Western Blotting

SKGT-4 cells were seeded at a density of 2 × 10^5^ cells per well in nunclon 6-well plates and allowed to adhere overnight. The following day after serum starving for 1 h the cells were treated with DCA (20 μM), DCA (200 μM), BA cocktail (100 μM) or PMA (100 ng/mL) for 6 h. The supernatants were collected and precipitated by mixing with equal volume of methanol and one quarter volume of chloroform before spinning at 20,000 rpm for 10 min at room temperature. The intermediate protein layer was mixed with 500 μL methanol, spun for 5 min at 20,000 rpm and once the supernatant was removed the pellet was dried at 55 °C for 10 min before being resuspended in 50 μL of 1× lamelli buffer. Afterwards, samples were loaded onto a SDS PAGE gel (25 μL per well) and Western blotting was performed as described previously. The PVDF membranes were incubated on a rocker overnight at 4 °C with MMP-10 primary antibody (1 in 500 in 5% BSA with NaN_3_) (Genetex, Irvine, CA, USA) and after washing with TBS-T were added to an anti-rabbit HRP secondary antibody (Sigma, St. Louis, MO, USA) in 5% marvel for 2 h. The membrane was developed and the MMP-10 protein was quantified as before using ImageJ software (NIH, Bethesda, MD, USA).

### 4.9. Immunohistochemical Staining and Analysis in Human Samples

Patients who were diagnosed with OAC were identified retrospectively from the pathology department records at the Mercy University Hospital, Cork. Ethical approval for this study was obtained from the Clinical Research Ethics Committee of the Cork Teaching. All of the patients were diagnosed and treated at the Mercy University Hospital, Cork between 2000 and 2006. Archival formalin fixed, paraffin embedded tumour blocks and corresponding normal oesophageal mucosa were collected. All tissues were from patients who did not receive neoadjuvant therapy (chemo-naive).

Normal squamous epithelial (*n* = 4) and OAC (*n* = 8) tissue sections were de-waxed through a serial grading of alcohols and antigen retrieval was performed using 0.1 M citrate buffer (pH 6.0) in a pressure cooker for 20 min and allowed to cool to room temperature. Endogenous peroxidase was blocked with 1% Hydrogen peroxide/PBS for 15 min at room temperature Slides were blocked with 1.5% normal goat serum (NGS)/PBS for 1 h to reduce nonspecific binding. Slides were incubated overnight at 4 °C with MMP10 antibody (GeneTexCat No. GTX113496) diluted 1:100 in 1.5% NGS/PBS. After washing in PBS, sections were incubated with 1:100 anti-rabbit secondary antibody (VECTASTAIN^®^ Elite^®^ ABC HRP Kit (Peroxidase, Rabbit IgG), Dako) at room temperature for 1 h. Slides were incubated with 3,3′-Diaminobenzidine tetrahydrochloride (DAB, Sigma Aldrich) for 5 min and sections were counterstained with Harris hematoxylin (Sigma-Aldrich). Slides were mounted with DPX and imaged using a Nikon microscope at 10× magnification. In negative control slides, NGS replaced the primary antibody.

A ranked tissue scoring system was used for the analysis of MMP10 staining with intensity as 0 (no stain), 1 (weak stain), 2 (moderate staining), 3 (strong staining) and % positivity as either 0, 25, 50, 75 or 100%. An Intensity × Positivity score was assigned for each tissue. At least 2 fields of view were obtained per patient tissue at 10× magnification.

### 4.10. Statistics

Statistical comparison between treatments was carried out using one sample t-tests or one-way ANOVA with Dunnett’s post-hoc correction with a *p* < 0.05 considered significant. In all cases, data are graphed as the mean ± standard error of the mean (SEM) with at least three separate replicates performed per experiment. All data were analysed using Graphpad Prism 5 (La Jolla, CA, USA).

## 5. Conclusions

The incidence of OAC is increasing in western countries despite the widespread use of PPIs, therefore low pH is not only the contributing factor. However, our understanding regarding the role of DCA in OAC development is still very limited. In our present work, we provide pioneering evidence that DCA promotes OAC invasiveness in vitro. As MMPs are enzymes that promote cell migration and cancer invasion, we next studied the effect of DAC on MMPs. We found that MMPs were over-expressed in DCA-treated OAC cells, in particular MMP-10 that correlates very well with the upregulation of MMP-10 in human OAC. This study does not show how DCA up-regulates MMP-10 in OAC; however, it will advance our understanding of the pathophysiological mechanisms involved in OAC and shows promise for the development of new therapeutic strategies.

## Supplementary Materials

The following are available online at https://www.mdpi.com/1422-0067/21/21/8042/s1.
