# Supplementary material for: Impact of Deoxycholic Acid on Oesophageal Adenocarcinoma Invasion: Effect on Matrix Metalloproteinases
Source: Int J Mol Sci. 2020 Oct 28;21(21):8042. doi: 10.3390/ijms21218042 (PMC7672620; doi:10.3390/ijms21218042)
Supplement: Supplementary file 1 [file ijms-21-08042-s001.pptx]

## Slide 1
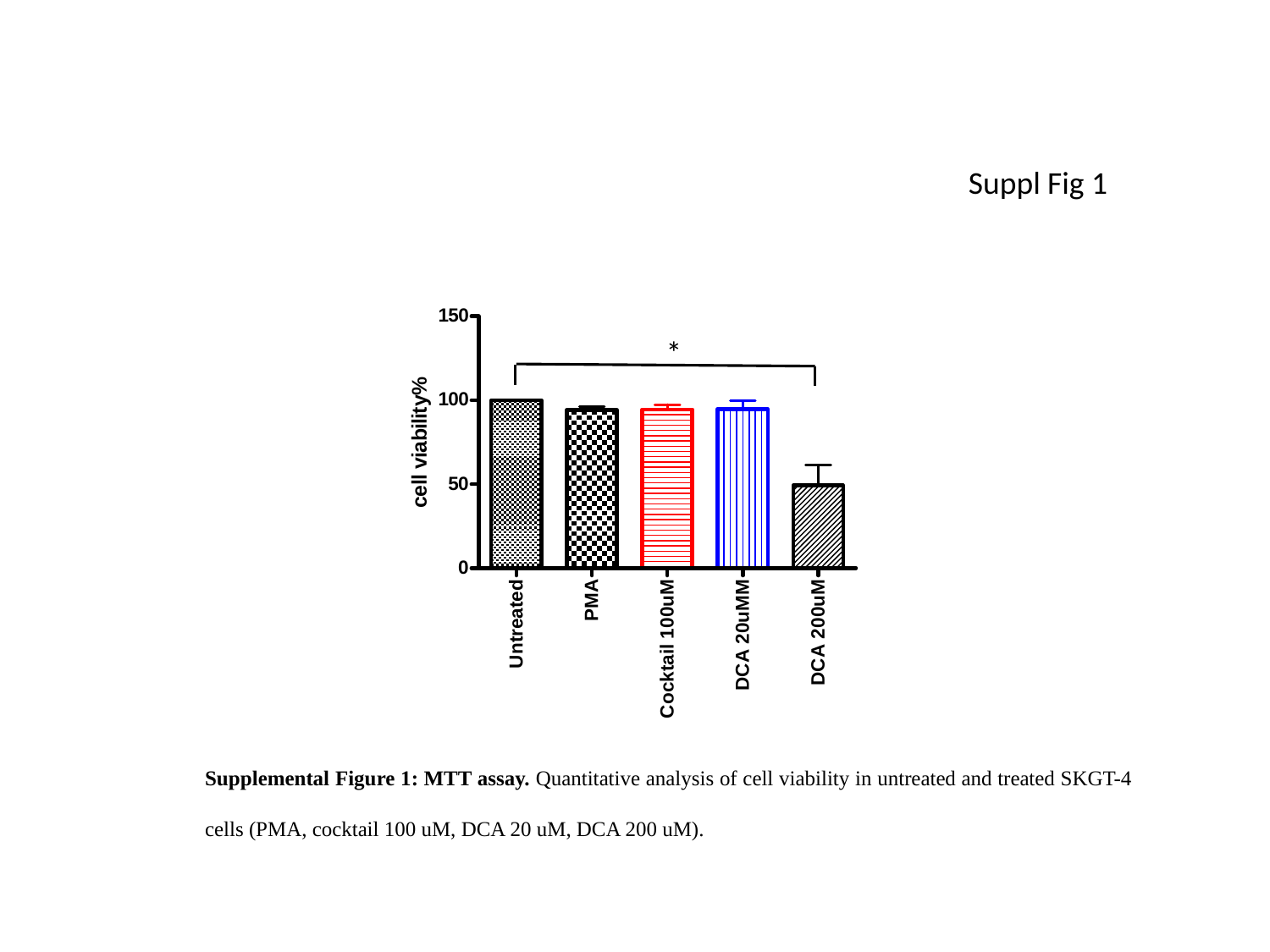

Suppl Fig 1
*
Supplemental Figure 1: MTT assay. Quantitative analysis of cell viability in untreated and treated SKGT-4 cells (PMA, cocktail 100 uM, DCA 20 uM, DCA 200 uM).
